# Synthesis of hierarchical metal nanostructures with high electrocatalytic surface areas

**DOI:** 10.1126/sciadv.adf6075

**Published:** 2023-01-11

**Authors:** Lucy Gloag, Agus R. Poerwoprajitno, Soshan Cheong, Zeno R. Ramadhan, Tadafumi Adschiri, J. Justin Gooding, Richard D. Tilley

**Affiliations:** ^1^ School of Chemistry, The University of New South Wales, Sydney, NSW 2052, Australia.; ^2^ Mark Wainwright Analytical Centre, The University of New South Wales, Sydney, NSW 2052, Australia.; ^3^ Institute of Multidisciplinary Research for Advanced Materials, Tohoku University, Sendai 980-8577, Japan.; ^4^ Advanced Institute of Materials Research, WPI-AIMR, Tohoku University, Sendai 980-8577, Japan.; ^5^ Australian Centre for NanoMedicine, The University of New South Wales, Sydney, NSW 2052, Australia.

## Abstract

3D interconnected structures can be made with molecular precision or with micrometer size. However, there is no strategy to synthesize 3D structures with dimensions on the scale of tens of nanometers, where many unique properties exist. Here, we bridge this gap by building up nanosized gold cores and nickel branches that are directly connected to create hierarchical nanostructures. The key to this approach is combining cubic crystal–structured cores with hexagonal crystal–structured branches in multiple steps. The dimensions and 3D morphology can be controlled by tuning at each synthetic step. These materials have high surface area, high conductivity, and surfaces that can be chemically modified, which are properties that make them ideal electrocatalyst supports. We illustrate the effectiveness of the 3D nanostructures as electrocatalyst supports by coating with nickel-iron oxyhydroxide to achieve high activity and stability for oxygen evolution reaction. This work introduces a synthetic concept to produce a new type of high-performing electrocatalyst support.

## Teaser

A multistep synthesis produces 3D nanomaterials with high surface areas, high conductivity and chemically tunable surfaces.

## INTRODUCTION

With the demand for smaller, more powerful technologies, scale is at the forefront of materials synthesis (*1*). At the molecular scale, bottom-up methodologies have been developed to successfully create three-dimensional (3D) networks of molecules, from nature-inspired DNA origami through to application-driven metal organic frameworks (*2*–*4*). At the micrometer scale, 3D foams with components that have widths of more than 100 nm have been successfully formed by top-down etching processes to create porous structures and aerogels (*5*, *6*). At the nanoscale, 3D superlattices made of ordered discrete nanoparticles coated in surfactant molecules have been successfully assembled (*7*–*9*). However, there is a noticeable gap for the synthesis of structures with nanosized metal components that are interconnected in 3D.

There is an important opportunity for such 3D nanostructures because they have the ideal properties for electrocatalytic support materials. First, the nanoscale dimensions create high surface areas, which ensures high exposure of the active catalyst to the reactants (*10*–*13*). Second, the directly connected metal components create a highly conductive material for efficient electron transfer between the catalysts and the electrode (*14*). Third, the metal surfaces can be chemically modified, which enables a catalytically active material to be coated onto the surface (*15*).

The formation of branched nanoparticles with two connected components is well known. For example, our group has shown that well-defined branches with dimensions on the tens of nanometers can be grown by carefully selecting a core material that adopts a face-centered cubic (fcc) structure, such as Pd or Au, and a branch material that adopts a hexagonal close-packed (hcp) structure, such as Ru, Co, or Ni (*13*, *16*–*20*). In these papers, the hcp branches preferentially grow along the *c* axis of the crystal structure to form elongated branches directly off the fcc cores. However, producing 3D interconnected structures on the nanoscale requires the development of a new multistep synthetic approach that builds multiple connected components in a hierarchical structure. To date, multistep processes have produced nanoparticles with several connected spherical components, such as trimers or oligomers (*21*, *22*). If fcc cores and hcp branches can be grown directly on each other in multiple sequential steps, then there is an opportunity to synthesize hierarchical 3D interconnected nanostructures.

In this work, we present a bottom-up synthetic approach that grows cores and branches in a multistep process to construct hierarchical 3D nanostructures. Using transmission electron microscopy (TEM), we show that by choosing fcc-Au as the core and hcp-Ni as the branch material, we can direct the growth into a hierarchical structure. We illustrate how controlling the reaction conditions at each step influences the 3D structure. By coating an oxygen evolution reaction (OER) catalyst nickel-iron oxyhydroxide [Ni/Fe-O(OH)] onto the surface of the 3D nanostructures, we show that this is an effective strategy to create materials that have a high surface area, are conductive, and have surfaces that can be chemically modified, which make high-performing electrocatalyst supports.

## RESULTS

### 3D nanostructure synthesis

In this work, we use Au as the fcc core material because it does not readily oxidize, which facilitates the direct growth of a second metal on its surface (*16*, *17*, *22*). We chose Ni as the branch material because it can form an hcp structure that grows into elongated branches (*16*). Ni is also an ideal metal for catalytic supports as shown by Ni nanofoams, which are one of the state-of-the-art electrode support materials (*15*, *23*–*25*).

**Fig. 1.**
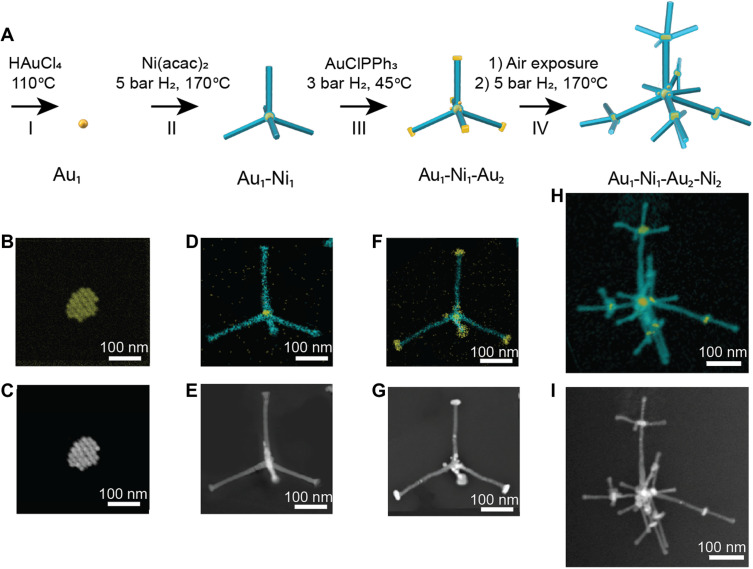
Multistep synthesis of 3D nanostructures. (**A**) Scheme illustrating the 3D structures of the nanoparticles synthesized at each synthetic step. Yellow, Au; cyan, Ni. (**B** to **I**) Scanning TEM (STEM)–energy-dispersive x-ray spectroscopy (EDX) maps and high-angle annular dark-field (HAADF)–STEM images of the nanoparticles after each step in the synthesis. (B and C) Au_1_ cores synthesized in step I. (D and E) Au_1_-Ni_1_ nanoparticle synthesized from Ni branch growth on the Au_1_ cores in step II. (F and G) Au_1_-Ni_1_-Au_2_ nanoparticle synthesized from Au core growth on the tips of Ni_1_ branches in step III. (H and I) Au_1_-Ni_1_-Au_2_-Ni_2_ 3D nanostructures synthesized from Ni branch growth on Au_2_ cores in step IV.

The approach for making Ni 3D nanostructures comprises four distinct steps, as illustrated in Fig. 1A. The products synthesized at each step were characterized using scanning TEM (STEM) coupled with energy-dispersive x-ray spectroscopy (EDX) and high-angle annular dark-field (HAADF)–STEM in Fig. 1 (B to I). In step I, 20-nm Au_1_ cores were formed by decomposing gold(III) chloride in a hot solution of toluene and oleylamine (Fig. 1, B and C, and fig. S1). In step II, Ni_1_ branches are grown on the Au_1_ cores by slowly reducing Ni(II) acetylacetonate using hydrogen gas. Hydrogen gas is used as a mild reducing agent because our previous work has shown that using H_2_ as a reducing agent facilitates heterogeneous growth of Ni directly onto Au (**16**). The Au_1_-Ni_1_ branched nanoparticles have four to six branches with widths of 20 nm (Fig. 1, D and E, and fig. S2). The branches extend in 3D, resulting in the appearance of width differences along the branch. Where the end facets are tilted toward the electron beam, there is the appearance of enlargement at the tips. All branch dimensions were measured for branches perpendicular to the electron beam. The branch width is similar to the diameter of the cores, which ensures that the 3D nanostructures have nanosized components.

In step III, Au_2_ cores are grown onto the tips of the Ni_1_ branches to form Au_1_-Ni_1_-Au_2_ nanoparticles (Fig. 1, F and G). The key synthetic consideration in this step is to ensure that Au grows on the tips rather than on the sides of the Ni branches. This was achieved by using very low concentrations of gold(I) chloride triphenylphosphine, which slowly decomposes in hydrogen gas (*26*). The slow deposition results in the nucleation of Au on the highly exposed tips of the Ni branches.

In step IV, Ni_2_ branches are grown on the Au_2_ to form Au_1_-Ni_1_-Au_2_-Ni_2_. The STEM-EDX map and HAADF-STEM image show the location of the Au cores in the center of the 3D nanostructures and at the tips of the Ni branches (Fig. 1, H and I). To grow Ni selectively on the Au_2_ cores, it is necessary to prevent growth on the sides of the Ni_1_ branches. This was achieved by exposing the Au_1_-Ni_1_-Au_2_ nanoparticles to air, which oxidizes and blocks the sides of the Ni branches (fig. S3). This oxidation process is important because without it, Ni_2_ preferentially grows on the sides of the branches rather than on the Au_2_ cores (fig. S4).

The stepwise growth of Au cores and Ni branches results in hierarchical nanostructures with 3D morphologies. The uniformity across the sample is shown by low-resolution scanning electron microscopy (SEM) imaging of Au_1_-Ni_1_-Au_2_-Ni_2_ nanostructures (fig. S5).

**Fig. 2.**
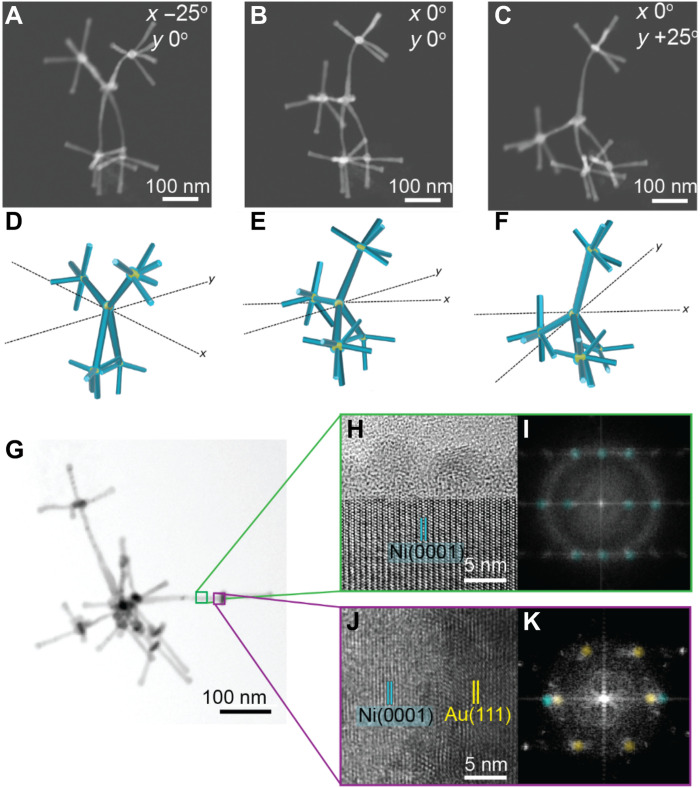
Characterization of the 3D nanostructures. (**A** to **C**) HAADF-STEM images that show the 3D nanoscale structure by tilting in the electron microscope at (A) −25° in the *x* plane, (B) 0°, and (C) +25° in the *y* plane. (**D** to **F**) Models illustrating the 3D structure at different tilt angles that match the images in (A) to (C). (**G**) Bright-field STEM image of a 3D nanostructure. (**H**) High-resolution TEM (HRTEM) of the Ni branch from the area in the green box in (G). The stacking of {0001} lattice planes of the Ni crystal occurs along the branch and is surrounded by a lighter contrast oxide that is partially crystalline. (**I**) Fast Fourier transform (FFT) of the image in (H). The spots match an hcp crystal structure from the Ni branch viewed down a <01-10> zone axis with a diffuse ring due to the oxide shell. (**J**) HRTEM of the interface between a Ni_1_ branch and Au_2_ core shown in the purple box in (G). Au lattice planes align epitaxially on top of Ni lattice planes. (**K**) FFT of the image in (J). Spots corresponding to Ni hcp(0001) viewed down a <01-10> zone axis and Au fcc(111) viewed down a <110> zone axis are aligned.

The 3D morphology was characterized using HAADF-STEM imaging over a series of tilt angles (Fig. 2, A to C). By tilting an individual nanostructure, the orientation of the branches alters relative to the electron beam. The images from the tilt series show the uniformity and 3D arrangement of branches. Models that match the HAADF-STEM images and that further illustrate the 3D structure are shown in Fig. 2 (D to F).

A typical Ni 3D nanostructure is shown in the bright-field STEM image in Fig. 2G, with the darker contrast in the image corresponding to the Au cores and the lighter contrast corresponding to the Ni branches. The SEM images in fig. S6 show that the Ni branches extend outward from the Au cores. The SEM images also show an increase in the number of Ni branches that extend from the central core, which results from additional Au_2_ cores forming on the central core that act as nucleation sites for Ni_2_ branch growth.

Evidence that the Ni branches grown in steps II and IV do adopt the hcp crystal structure is shown in the high-resolution TEM (HRTEM) image and fast Fourier transform (FFT) of the image (Fig. 2, H and I). The close-packed {0001} planes are arranged in an ABAB sequence in the direction of branch propagation, indicating that the branches grow along the *c* axis of the hcp crystal. This growth along the *c* axis is vital for the formation of 3D and crystalline branched metal nanoparticles (*16*, *20*). The surface Ni oxide formed on the sides of Ni branches is also observable as a lighter contrast polycrystalline layer on the side of the branch in the TEM image in Fig. 2 (H and I). The Ni oxide is formed after minutes of exposure in air. The Ni oxide layer is important in step IV to direct Ni growth onto the Au cores due to the preferential formation of metal-metal bonds (*22*).

The interface between a Ni branch and Au core in the HRTEM image in Fig. 2J shows that there is a change in stacking sequence from ABAB for hcp-Ni to ABCABC for fcc-Au. The FFT of the HRTEM image shows that the spots corresponding to the fcc{111} lattice planes of the Au (yellow) overlap with the spots corresponding to the hcp{0001} lattice planes of Ni (cyan, Fig. 2K). These results indicate that the hcp-Ni and fcc-Au are crystallographically aligned, and the growth of the Au core occurs epitaxially off the Ni branch. As shown by the presence of both fcc and hcp Ni in the x-ray diffraction pattern (fig. S7), the Ni_1_ shell is polycrystalline, which results in some Au_2_ cores that are also observed at the center of the nanoparticles.

**Fig. 3.**
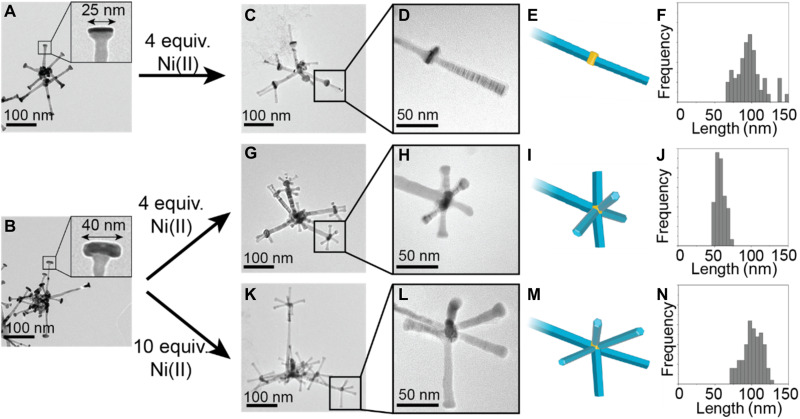
Controlling the 3D nanostructure. (**A**) TEM image of Au_1_-Ni_1_-Au_2_ nanoparticles formed from one reaction of Au precursor. (**B**) TEM image of Au_1_-Ni_1_-Au_2_ nanoparticles formed from a second reaction of Au precursor with the nanoparticles shown in (A). (**C**) 3D nanostructure from the reaction of Ni precursor with the Au_1_-Ni_1_-Au_2_ nanoparticles with a 25-nm Au_2_ core shown in (A) using 4 equivalents of Ni precursor relative to Au_2_. (**D**) Single Ni_2_ branch grown from a 25-nm Au_2_ core from the nanoparticle in (C). (**E**) Illustration of a single Ni_2_ branch grown off a 25-nm Au_2_ core. (**F**) Frequency plot of the number of Ni_2_ branches per Au_2_ core for the 3D nanostructures shown in (B). (**G**) 3D nanostructure formed from the reaction of Ni precursor with the Au_1_-Ni_1_-Au_2_ nanoparticles with a 40-nm Au_2_ core shown in (B) using 4 equivalents of Ni precursor relative to Au_2_. (**H**) Multiple Ni_2_ branches grown from a 40-nm Au_2_ core from the nanoparticle in (B). (**I**) Illustration of multiple, short Ni_2_ branches grown off a 40-nm Au_2_ core. (**J**) Histogram of the Ni_2_ branch length for the 3D nanostructures shown in (G). (**K**) 3D nanostructure formed from the reaction of Ni precursor with the Au_1_-Ni_1_-Au_2_ nanoparticles with a 40-nm Au_2_ core shown in (B) using 10 molar equivalents of Ni precursor relative to Au_2_. (**L**) Multiple Ni_2_ branches grown from a 40-nm Au_2_ core from the nanoparticle in (B). (**M**) Illustration of multiple, long Ni_2_ branches grown off a 40-nm Au_2_ core. (**N**) Histogram of the Ni_2_ branch length for the 3D nanostructures shown in (K).

An advantage of this multistep synthetic approach is that it enables control over the dimensions and 3D structure (Fig. 3 and Table 1). The size of the Au_2_ cores can be controlled by tuning the amount of AuClPPh_3_ deposited on the Ni_1_ tips (fig. S8). When a reaction of 1:5 AuClPPh_3_:Ni_1_ was performed, Au_2_ cores with a diameter of 25 nm formed (Fig. 3A and fig. S8A). When this reaction was repeated a second time, the Au_2_ cores grew to 40 nm in diameter (Fig. 3B and fig. S8B).

**Table 1.** Quantities of Au_1_-Ni_1_-Au_2_ particles used in the preparation of 3D nanostructures.

	Ratio of Ni(II) to Au_2_	Au_2_ in total Au_1_-Ni_1_-Au_2_ sample	Proportion of Au_1_-Ni_1_-Au_2_ sample used
3D nanostructures - single 100-nm Ni_2_	4:1	0.006 mmol	0.20
3D nanostructures - multiple 58-nm Ni_2_	4:1	0.012 mmol	0.10
3D nanostructures - multiple 100-nm Ni_2_	10:1	0.012 mmol	0.04

The number of branches extending from the tips (number of Ni_2_ branches) can then be controlled using the different sized Au_2_ cores. When 25-nm Au_2_ cores were used, only one Ni branch formed (Fig. 3, B to D, and fig. S9). When 40-nm Au_2_ cores were used, 3D nanostructures with an average of five Ni_2_ branches were formed (Fig. 3, F to H, and figs. S10 and S11). This indicates that the Au_2_ cores must have a large enough surface area to accommodate multiple branches.

The length of the Ni_2_ branches can also be controlled by tuning the ratio of Ni precursor:Au_2_. The amount of Au_2_ in the Au_1_-Ni_1_-Au_2_ nanoparticles was calculated on the basis of the molar quantities of AuClPPh_3_ precursor used in step III (see the “Experimental design” section). The length of the Ni_2_ branches was increased from 58 to 100 nm by tuning the ratio of Ni(II):Au_2_ between 4:1 and 10:1 (Fig. 3, I to K). By keeping the concentration of Ni(acac)_2_ precursor constant and decreasing the concentration of Au cores, the amount of Ni per core is increased while preventing homogeneous nucleation of separate Ni nanoparticles.

### Electrocatalyst support properties

**Fig. 4.**
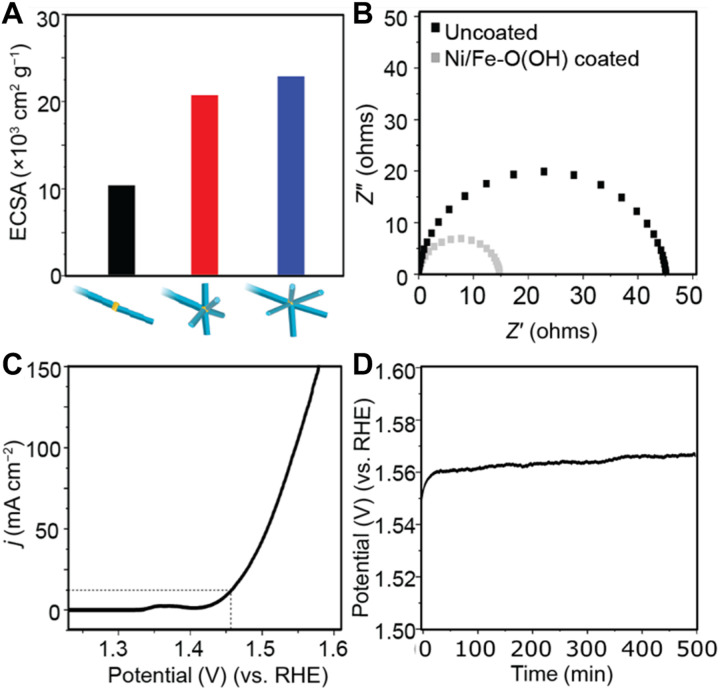
Properties of 3D nanostructures as electrocatalytic supports. (**A**) Electrochemically active surface area (ECSA) of 3D nanostructures with a single 100-nm Ni_2_ branch (black), multiple 58-nm Ni_2_ branches (red), and multiple 100-nm Ni_2_ branches (blue). (**B**) Electrochemical impedance spectra (EIS) before and after coating the Ni branches with the material Ni/Fe-O(OH) layer. (**C**) OER polarization curve showing the current density (*j*) of Ni/Fe-O(OH)–coated 3D nanostructure supports in 0.1 M KOH. Dotted line shows the potential needed to reach a current density of 10 mA cm^−2^. (**D**) Chronopotentiometric scan showing the potential required to maintain a constant current density of 10 mA cm^−2^ over time.

To characterize the physical and electrochemical properties of the 3D nanostructures, electrodes were prepared from the nanoparticles shown in Fig. 3 (C, G, and K) by drop-casting onto a rotating-disk electrode with a loading of 15 μg (see the “Experimental design” section and figs. S12 and S13). The 3D nanostructures with multiple Ni_2_ branches have electrochemically active surface areas (ECSAs) that are two times greater than nanostructures with a single Ni_2_ branch (Fig. 4A and table S1). The 3D nanostructures with multiple 100-nm Ni_2_ branches have a slightly increased ECSA compared to the 3D nanostructures with multiple 58-nm Ni_2_ branches. This verifies that multiple branches give higher ECSA and that the number of branches is an important structural feature for increasing the ECSA. The nanoscale dimensions of the 3D nanostructures create supports with very high ECSAs with 3D nanostructures having an ECSA of up to 100 times greater than state-of-the-art nickel foam supports (*15*). The nanoscale dimensions of the 3D nanostructures bridge the gap between the micrometer and molecular scales, with the ECSA per mass being in between microscale Ni foams and Ni clusters (table S1).

To illustrate the effectiveness as electrocatalyst supports, we loaded the 3D nanostructures with an electrocatalyst material and examined the performance. The OER active material nickel-iron oxyhydroxide [Ni/Fe-O(OH)] was chosen as the electrocatalyst as its performance on Ni foam electrocatalyst supports has been comprehensively reported (*15*, *27*–*29*). To adequately assess the role of the 3D nanostructure support, Ni/Fe-O(OH) was deposited via a well-established electrodeposition method (see the “Experimental design” section) (*15*, *27*–*29*). STEM-EDX imaging shows that a Ni/Fe-O(OH) deposits uniformly on the surface of the 3D nanostructure (fig. S14). A maximum current density for OER was achieved when Ni/Fe-O(OH) was electrodeposited for 75 s (fig. S15), indicating that this deposition time generates sufficient active sites on the supports. The charge transfer resistance determined from electrochemical impedance spectroscopy (EIS) decreased from 44 to 15 ohms after coating with Ni/Fe-O(OH) for 75 s (Fig. 4B). This decrease has been previously observed when there is effective contact between the nickel support and the Ni/Fe-O(OH) (*30*, *31*). Low charge transfer resistance values between 44 and 53 ohms were observed for all 3D nanostructures before catalyst deposition (fig. S16). This illustrates that directly growing conductive Au and Ni metals into 3D nanostructures creates highly conductive supports that match microscale Ni foams (table S1).

The OER performance of the Ni/Fe-O(OH)–coated 3D nanostructures was evaluated in 0.1 M KOH (Fig. 4C). A geometric current density of 10 mA cm^−2^ was reached at a low overpotential of 225 mV. This is 15 mV lower than that of state-of-the-art Ni/Fe-O(OH)–coated microscale Ni foams under the same conditions (table S2). A specific activity of 4.2 mA cm_ECSA_^−2^ was achieved, which is lower than that of state-of-the-art Ni/Fe-O(OH) catalysts (*15*, *27*–*29*). This indicates that the high geometric current density results from the high surface area of the 3D nanostructure support rather than any intrinsic properties of the catalyst material.

The stability of the Ni/Fe-O(OH)–coated 3D nanostructures was assessed by chronopotentiometry at a current density of 10 mA cm^−2^ (Fig. 4D). After 10 min, a steady-state potential of 1.56 V was reached, and this was maintained over 500 min. The OER polarization curves show only a small decrease in the current density of 20% (fig. S17), which indicates that they are stable for OER. TEM analysis performed after chronopotentiometry shows that the morphology of the 3D nanostructures is unchanged (fig. S18), indicating that the supports are structurally stable. The high electrocatalytic performance originates from the high ECSA, high conductivity, and effective surface modification and illustrates the advantage of using 3D nanostructures as electrocatalytic supports.

## DISCUSSION

In this work, we illustrate a bottom-up synthetic concept to form 3D nanostructures with nanoscale dimensions that bridge the gap between molecular and micrometer-sized 3D materials. We construct the 3D structure by directly growing core and branch components in multiple controlled steps. In this synthetic approach, the choice of materials is important, an hcp material is needed to grow into elongated branches that extend from the cores to form 3D structures, and an fcc material is needed for the core that can be grown selectively on the tips of the branches. By using fcc crystal–structured Au cores and hcp crystal–structured Ni branches, we illustrate that the approach allows the tuning of reaction conditions at each step to achieve 3D nanostructures with single and multiple branches of widths of 20 nm and lengths between 50 and 100 nm.

Unique chemical and physical properties result from the 3D interconnected nanostructure. The materials have a high surface area as a result of the 3D and nanoscale structure. They are also highly conductive due to the direct connection of metallic cores and branches. The surfaces can be chemically modified by a catalytically active material. The effectiveness as electrocatalyst support materials is shown by the high OER activity and stability of Ni/Fe-O(OH)–coated 3D nanostructures. This controlled, multistep synthesis to construct hierarchical 3D nanostructures is a valuable approach to unlock 3D materials on the tens of nanometer scale and generate materials with unique catalytic, magnetic, and electronic properties.

## MATERIALS AND METHODS

### Experimental design

The 3D nanostructures were synthesized by sequential growth of Au and Ni blocks, and their properties were examined using electrochemical analysis.

#### Synthesis of Au_1_ seeds

Gold chloride trihydrate (12.5 mg; 99%; Sigma-Aldrich) was mixed with 0.3 ml of toluene and 0.3 ml of oleylamine (70%; Sigma-Aldrich). This solution was injected to 12.5 ml of toluene and 0.75 ml of oleylamine at 110°C. The temperature was maintained for 50 min. Then, a solution of 25 mg of gold chloride trihydrate mixed with 4 ml of toluene and 0.5 ml of oleylamine was injected into the reaction mixture at a rate of 0.1 ml min^−1^. The reaction was left for 1 hour before cooling to room temperature. The product was collected by centrifugation at 7800 rpm for 15 min.

#### Synthesis of Au_1_-Ni_1_ nanoparticles

Nickel(II) acetylacetonate (81 mg, 0.3 mmol, 95%; Sigma-Aldrich), Au seeds (2.4 mg, 0.07 mmol), hexadecylamine (1.44 g, 6 mmol, 98%; Sigma-Aldrich), and trioctylphosphine (70 μl, 0.18 mmol, 97%; Sigma-Aldrich) were dissolved in 20 ml of mesitylene. The solution was stirred and then transferred into a Parr reactor (nonstirred pressure vessel 4766 from Parr Instrument Company). The reactor was filled with 5 bar of hydrogen gas and placed in an oil bath at 170°C. After 24 hours, the reactor was cooled down, and the hydrogen was released. The nanoparticles were separated by magnetic force: A neodymium disk (15 mm) was placed beside a vial containing the nanoparticle solution and left for 5 min. The magnetic nanoparticles were collected at the side of the vial, and the colorless supernatant was decanted. The nanoparticles were redispersed in toluene in a glove box filled with Ar gas.

#### Synthesis of Au_1_-Ni_1_-Au_2_ nanoparticles

Hexadecylamine (7.6 mg, 0.03 mmol), AuClPPh_3_ (3.2 mg, 0.006 mmol, 99%; ChemSupply), and Au-Ni nanoparticles (0.03 mmol of Ni) were dispersed in 2 ml of toluene in a glove box filled with Ar gas. The reaction was sonicated, filled with 3 bar of hydrogen gas, and placed in an oil bath at 45°C for 24 hours. The solution was cooled, and the product was separated by magnetic force. All AuClPPh_3_ was reacted as indicated by a colorless supernatant. For the second 40-nm Au cores, the process was repeated.

#### Synthesis of Au_1_-Ni_1_-Au_2_-Ni_2_ 3D nanostructures

The sides of the Ni branches were oxidized by exposing the Au_1_-Ni_1_-Au_2_ nanoparticles to air. The reaction solution was then prepared in air. A solution (0.5 ml) containing nickel(II) acetylacetonate (3.0 mg ml^−1^, 0.01 mmol ml^−1^), hexadecylamine (26 mg ml^−1^, 0.1 mmol ml^−1^), and trioctylphosphine (2.4 μl ml^−1^, 0.006 mmol ml^−1^) was added to the Au_1_-Ni_1_-Au_2_ nanoparticles. The portion of Au_1_-Ni_1_-Au_2_ nanoparticles was used to make a solution with a controlled molar ratio of Ni(II) precursor to Au_2_, outlined in Table 1. The amount of Au_2_ was based on the molar quantity of AuClPPh_3_ used in the synthesis of Au_1_-Ni_1_-Au_2_ nanoparticles in the previous step. The solution was stirred, placed in a Parr reactor, filled with 5 bar of hydrogen gas, and placed in an oil bath at 170°C for 24 hours. The reactor was cooled, and hydrogen was released. The nanoparticles were separated by magnetic force and redispersed in toluene. The entire process of synthesizing Au-Ni-Au-Ni nanostructures was performed over 5 days. Three batches were produced simultaneously for each reaction to ensure reproducibility.

#### Electron microscopy

TEM, STEM, and STEM-EDX were performed on a JEOL JEM-F200 operated at 200 kV with a cold field-emission gun and equipped with an annular dark-field detector and a JEOL windowless 100-mm^2^ silicon drift x-ray detector. SEM images were taken on a FEI (Thermo Fisher Scientific) Nova NanoSEM 450.

#### ICP-MS characterization

For the inductively coupled plasma mass spectrometry (ICP-MS), the samples were digested in aqua regia and analyzed using a PerkinElmer Nexion instrument. All nanoparticle concentrations were measured by ICP-MS with an accuracy of 0.02 mg liter^−1^.

#### Electrochemical measurements

The electrochemical measurements were carried out in a three-electrode system using 0.1 M KOH as the electrolyte (pH 13.0) with Pt mesh as counter electrode and 1 M Hg|HgO|NaOH as reference electrode. To prepare the electrode, a solution of 1 mg_Ni_ ml^−1^ of nanoparticles in toluene was drop-casted onto a glassy carbon electrode to give a mass loading between 5 and 25 μg. Surfactant was electrochemically removed from the nanoparticles by running 20 CVs between 1.0 and 1.3 V [versus reversible hydrogen electrode (RHE)] at a scan rate of 20 mV s^−1^. The ECSA was calculated from the capacitive current at different scan rates. Cyclic voltammetry measurements in the capacitive potential range were performed at 5, 10, 20, and 50 mV s^−1^. The capacitance was determined from the slope of the capacitive current versus scan rate plots and then used to calculate the ECSA of the bare 3D nanostructures before electrocatalyst loading using the specific capacitance of Ni (300 μF cm^−2^) (*32*). EIS was performed with a Solartron SI 1287 electrochemical interface and SI 1260 impedance/gain-phase analyzer in a frequency range of 10^5^ to 10^−1^ and ac amplitude of 10 mV. The EIS measurement was performed at 1.60 V (versus RHE). The fitting for EIS spectra was done using one time constant [*R*(*QR*)] using the “ZSimp.Win 3.22” software. The Ni/Fe-O(OH)–coated catalysts were prepared by electrodeposition using an electrolyte solution containing 3 mM Ni(NO_3_)_2_·6H_2_O and 3 mM Fe(NO_3_)_3_·9H_2_O, as reported previously (*15*). Electrodeposition was performed at −1.0 V (versus Ag/AgCl) at 10°C between 30 and 120 s. The electrode was then rinsed with water and ethanol and dried in air. OER activity was assessed by measuring CVs between 1.2 and 1.7 V versus RHE with a scan rate of 5 mV s^−1^. The ECSA and OER activity of each nanoparticle sample were analyzed three times by preparing a new electrode and repeating the experimental procedures outlined above. All reported currents are corrected with respect to the capacitance in the CV and the uncompensated resistance (iR drop) with the resistance determined by EIS at open-circuit potential. OER stability was assessed by chronopotentiometry measured at a geometric current density of 100 mA cm^−2^ for 3 hours. All potentials are reported relative to the RHE unless otherwise specified according to equation

$$E_{\mathrm{RHE}}=E_{\mathrm{Hg}/\mathrm{HgO}}+0.059\mathrm{pH}+{E^{0}}_{\mathrm{Hg}/\mathrm{HgO}}$$

## Supplementary Materials

### This PDF file includes:

Supplementary Text Figs. S1 to S18 Tables S1 and S2 References
