# Supplementary material for: Synthesis of hierarchical metal nanostructures with high electrocatalytic surface areas
Source: Sci Adv. 2023 Jan 11;9(2):eadf6075. doi: 10.1126/sciadv.adf6075 (PMC9833653; doi:10.1126/sciadv.adf6075)
Supplement: Supplementary file 1 — Supplementary Text Figs. S1 to S18 Tables S1 and S2 References [file sciadv.adf6075_sm.pdf]

Science Advances
AAAS

Supplementary Materials for

Synthesis of hierarchical metal nanostructures with high electrocatalytic surface areas

Lucy Gloag *et al.*

Corresponding author: Richard D. Tilley, r.tilley@unsw.edu.au

Sci. Adv. **9**, eadf6075 (2023)
DOI: 10.1126/sciadv.adf6075

This PDF file includes:

Supplementary Text
Figs. S1 to S18
Tables S1 and S2
References

Supplementary Text

Distance between planes of Au(111) facet = d [Au(111)] = 2.355 Å

Distance between planes of Ni(0001) facet = d [Ni(01$\bar{1}$1)] = 2.033 Å

Lattice mismatch of Ni(0001) growth on Au(111) = 14 %

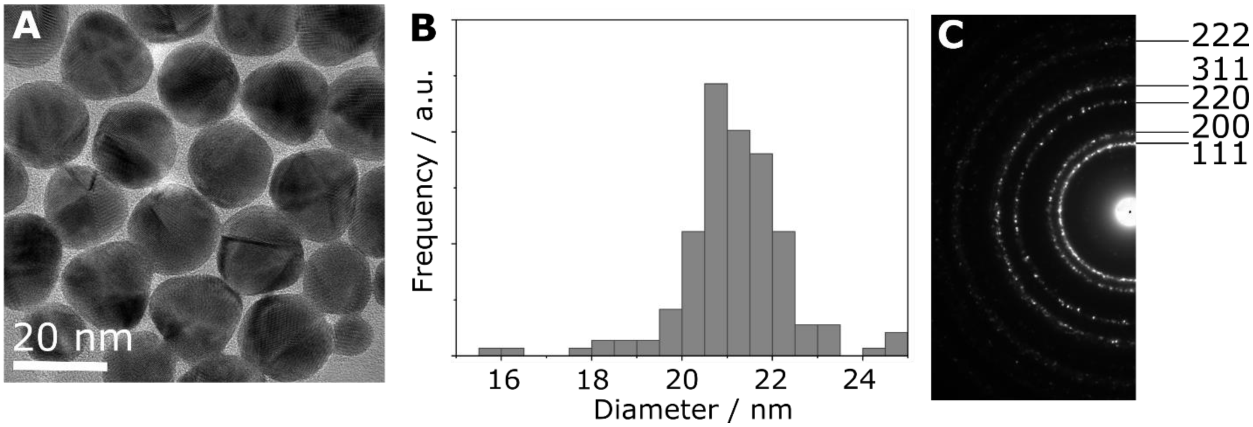

Fig. S1.

Au$_1$ nanoparticle cores. (**A**) HRTEM image showing nanoparticles with crystalline lattice fringes. (**B**) Histogram of the Au$_1$ nanoparticles shown in (A). (**C**) Diffraction pattern of the Au1 nanoparticles shown in (A) with rings indexed to an Au fcc crystal structure.

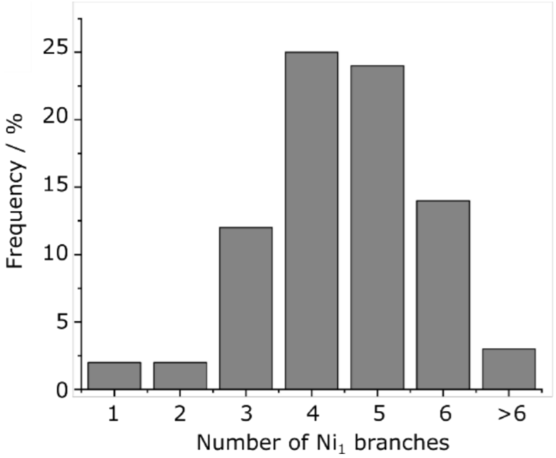

Fig. S2.

Plot of number of Ni1 branches per Au_1 core.

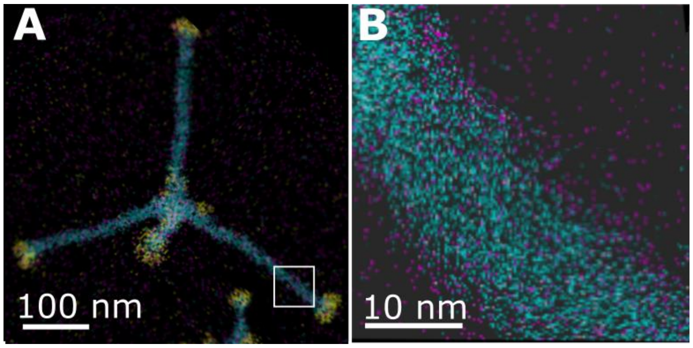

Fig. S3.

Ni oxide layer. (**A**) STEM-EDX map of a Au_1-Ni_1-Au_2 nanoparticle. (**B**) STEM-EDX map of the area in the box in (A) showing a layer of O around the Ni branch. Yellow, Au. Cyan, Ni. Magenta, O.

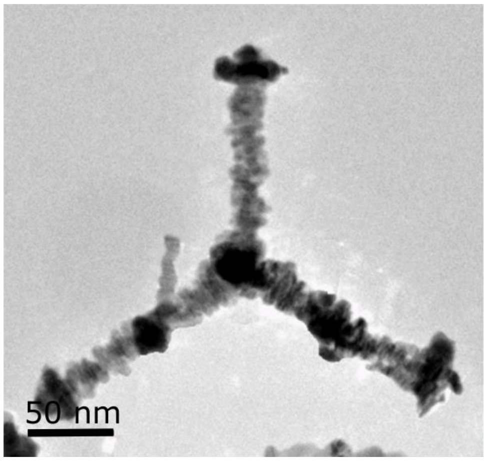

Fig. S4.

Ni growth without oxidation of Au_1-Ni_1-Au_2 nanoparticles. Ni_2 deposited on the sides of Ni_1 branches.

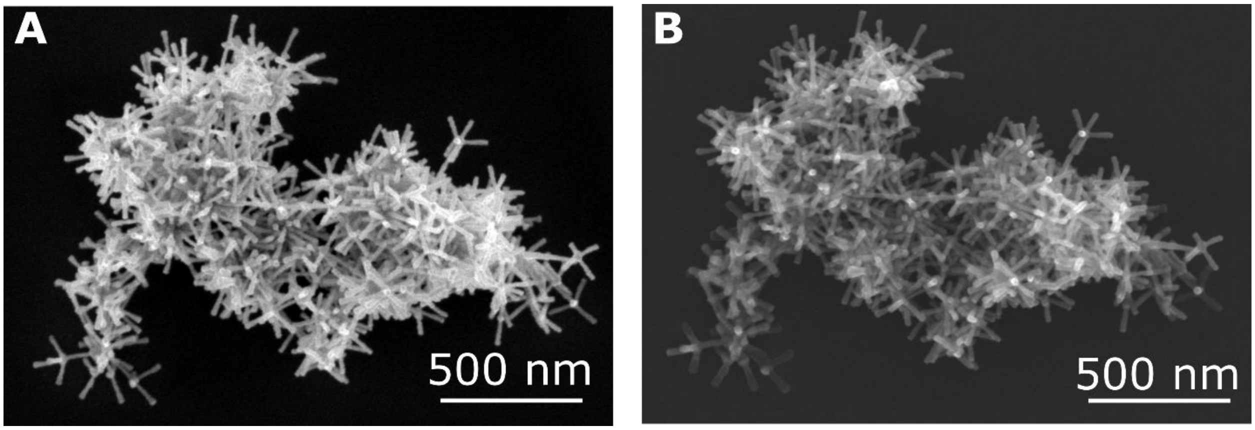

Fig. S5

Low resolution SEM of Au_1-Ni_1-Au_2-Ni_2 nanoparticles. (A) Back scattered electron SEM image. **(B)** Secondary electron SEM image.

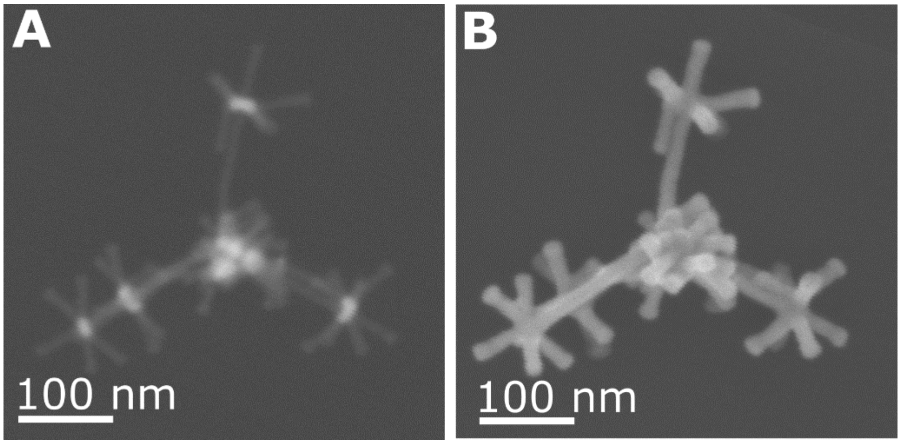

Fig. S6.

SEM images of a 3D nanostructure. (**A**) Back scattered electron SEM showing the brighter contrast Au and darker contrast Ni. (**B**) Secondary electron SEM image showing the 3D morphology.

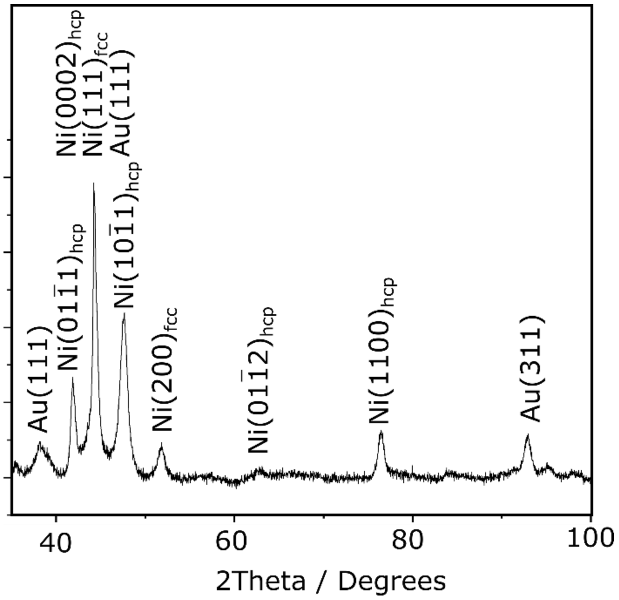

Fig. S7.

XRD of Au_1-Ni_1 nanoparticles. The peaks match with patterns from hcp-Ni, fcc-Ni and fcc-Au.

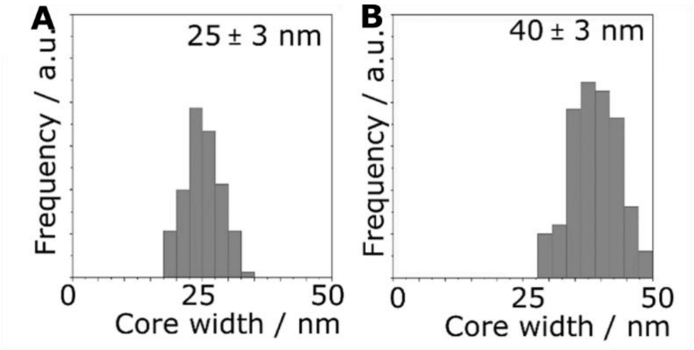

Fig. S8.

Histograms of Au_2 size. Histograms of the Au_2 size formed from (**A**) 1 reaction of Au precursor with the Au_1-Ni_1 nanoparticles and (**B**) a second reaction of Au precursor.

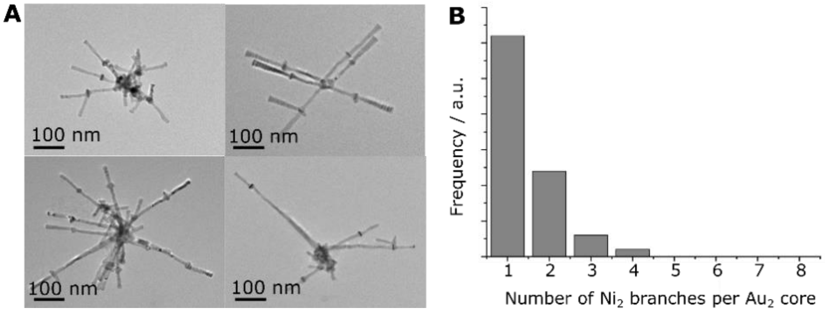

Fig. S9.

3D nanostructures with single Ni_2 branches. (**A**) TEM images of typical nanoparticles formed from the reaction of 4 equivalents of Ni precursor with Au_1-Ni_1-Au_2 nanoparticles with a 25 nm Au_2 core. (**B**) Plot of the number of Ni_2 branches per Au_2 core determined from counting over 100 Au_2 cores.

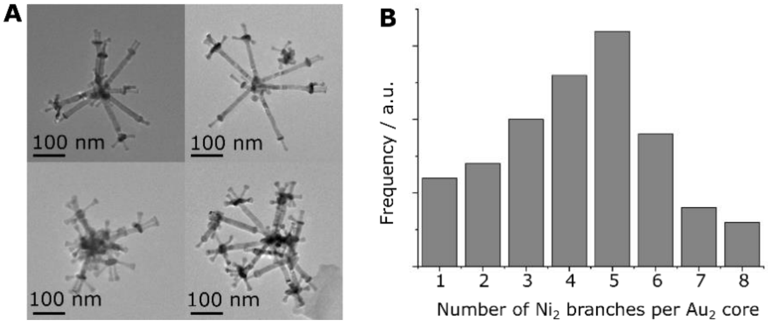

Fig. S10.

3D nanostructures with multiple short Ni_2 branches. (**A**) TEM images of typical nanoparticles formed from the reaction of 4 equivalents of Ni precursor with Au_1-Ni_1-Au_2 nanoparticles with a 40 nm Au_2 core. (**B**) Plot of the number of Ni_2 branches per Au_2 core determined from counting over 100 Au_2 cores.

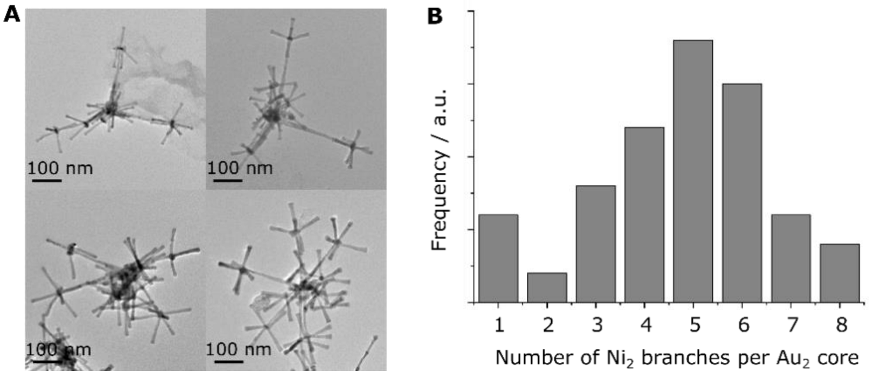

Fig. S11.

3D nanostructures with multiple long Ni_2 branches. (**A**) TEM images of typical nanoparticles formed from the reaction of 10 equivalents of Ni precursor with Au_1-Ni_1-Au_2 nanoparticles with a 40 nm Au_2 core. (**B**) Plot of the number of Ni_2 branches per Au_2 core determined from counting over 100 Au_2 cores.

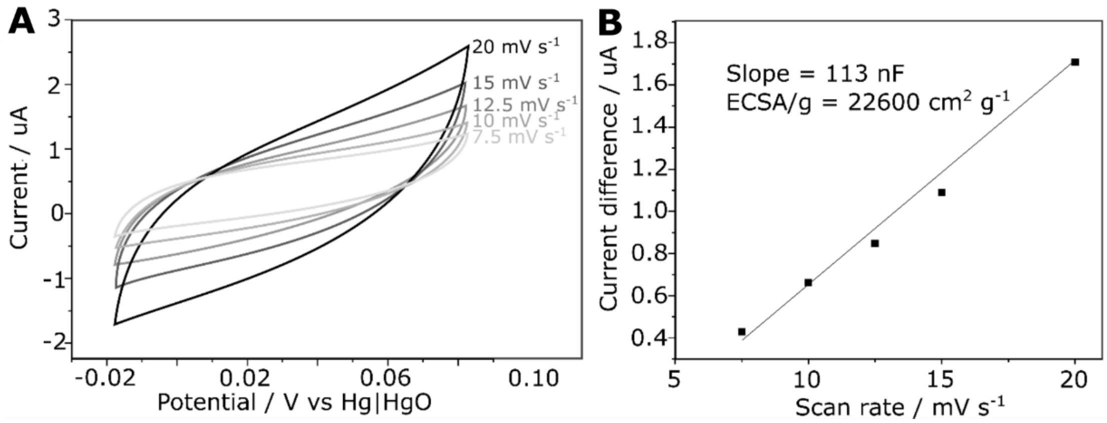

Fig. S12.

Calculation of electrochemically active surface areas. (**A**) Cyclic voltammograms in the non-faradaic region at different scan rates of the electrode loaded with 15 ug 3D nanostructures with multiple 100 nm branches. (**B**) Plot of capacitive current vs scan rate used to calculate the ECSA of 22600 cm^2 g^{-1}.

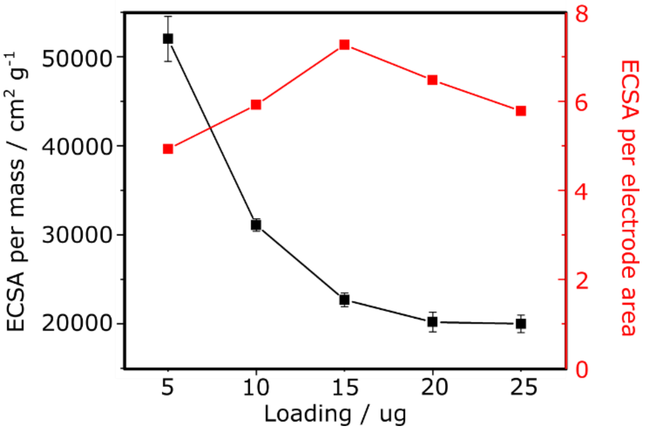

Fig. S13.

ECSA obtained with different loadings of nanoparticles on the electrode. Black line shows the ECSA per mass and red shows the ECSA per area of electrode (roughness factor).

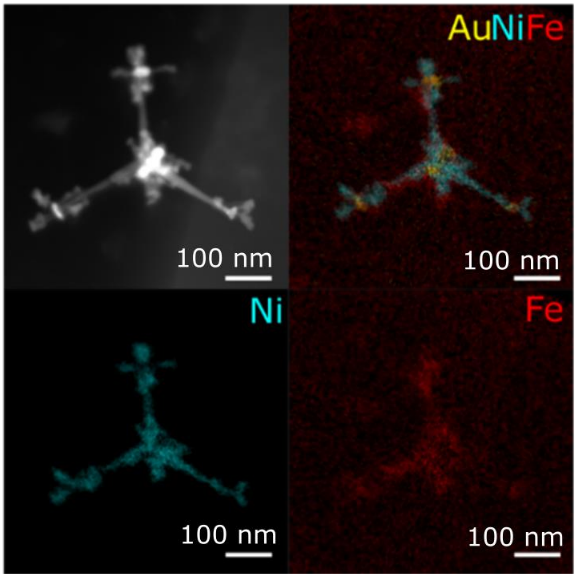

Fig. S14.

Ni/Fe-O(OH) coated 3D nanostructures. HAADF-STEM and EDX maps of a 3D nanostructure loaded with Ni/Fe-O(OH). The TEM grid was loaded with 1µg nanostructures (15x less than on the electrode) and deposited with Ni/Fe-O(OH) for 5s (15x less than on the electrode).

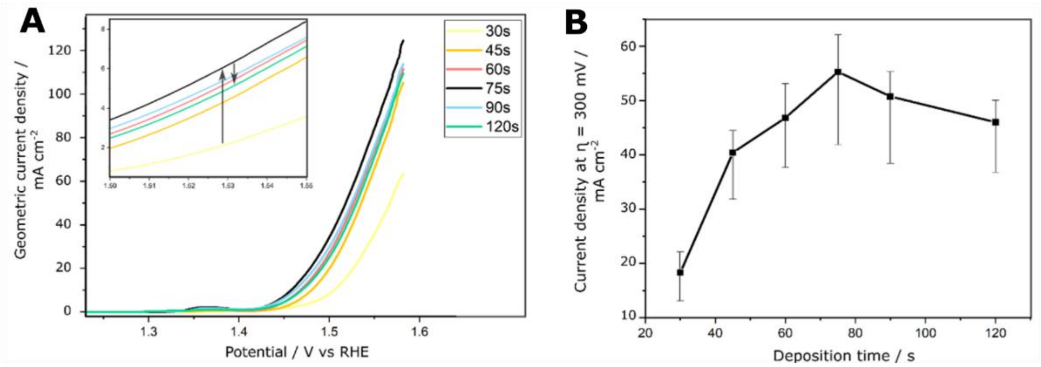

Fig. S15.

OER performance with different Ni/Fe-O(OH) deposition times. (**A**) OER polarization curves for 3D nanostructures electrodeposited with Ni^{2+}/Fe^{2+} for 30-120s. Inset shows the changes in current density between 1.50-1.56 V (vs RHE). (**B**) Plot showing the current density vs deposition time.

The current density generated at all potentials increased as the deposition time of Ni/Fe-O(OH) was increased up to 75 s. Deposition for longer than 75 s resulted in lower current densities as the Ni/Fe-O(OH) film becomes too thick and are not fully exposed for catalytic reaction. The 75 s deposition time is significantly shorter than the deposition time for Ni foams (300s) as the smaller dimensions of the 3D nanostructures require thinner films to optimally coat the surface.

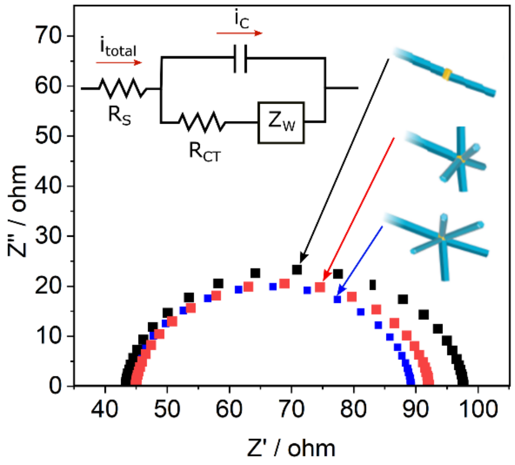

Fig. S16.

Electrochemical impedance spectroscopy. Nyquist plots for 3D nanostructures with multiple 100 nm Ni_2 branches (blue), multiple 58 nm Ni_2 branches (red) and single 100 nm Ni_2 branches (black). Inset shows an illustration of the Randles equivalent circuit used to represent a simple electron-transfer reaction.

This system fits well with a Randles equivalent circuit as shown by all measurements producing a single perfect semi-circle. Because water is the reactant in OER, we do not have Warburg resistance (Z_W). The resistance of the solution (Rs) remained consistent for all measurements between 43.5-45 ohm.

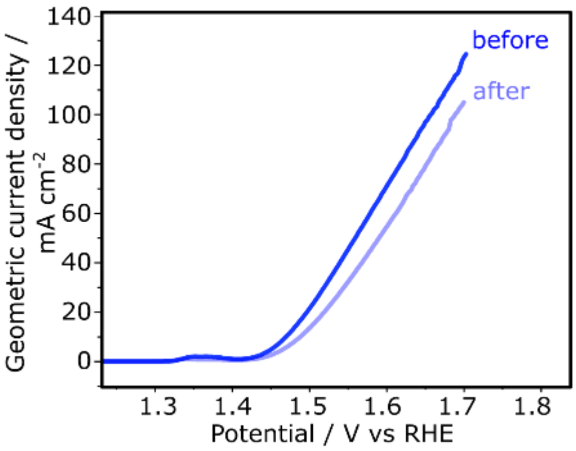

Fig. S17.

OER stability. Cyclic voltammograms before and after 180 mins chronopotentiometry at a constant current of 10 mA cm^{-2} for Ni/Fe-O(OH) coated 3D nanostructures.

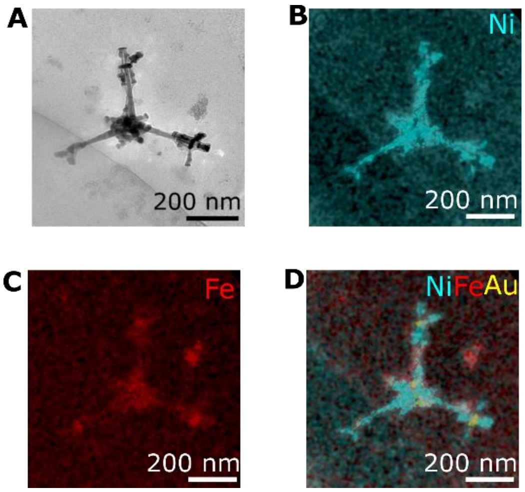

Fig. S18.

Post-catalysis TEM. TEM image of Au-Ni-Au-Ni nanostructures after 500 mins chronopotentiometry at a constant current of 10 mA cm^{-2}. (**A**) TEM image. (**B-D**) STEM-EDX images with Ni in cyan, Fe in red and Au in yellow.

Table S1.
Comparison of electrocatalyst support properties.

	**ECSA / $cm^2\ g_{Ni}^{-1}$**	**R_{CT} / ohm at 1.6 V**	**Reference**
**3D nanostructures -multiple 100 nm Ni_2**	22,600 ± 1,000	44	This work
**3D nanostructures -multiple 58 nm Ni_2**	20,500 ± 800	46	This work
**3D nanostructures -single 100 nm Ni_2**	10,400 ± 600	53	This work
**Ni Foam**	227	20	(*6*)
**Ni Foam**		35	(*24*)
**UltraSmall Ni NPs**	2,100,000		(*33*)
**Etched Ni Foam**		18	(*6*)
**Reduced Ni Foam**		33	(*6*)
**Bulk Ni**		211	(*6*)
**Ni nanoparticles**		36.7	(*34*)

Table S2.

Comparison OER performance to other supported Ni/Fe-O(OH) catalysts.

	**Overpotential to reach 10 mA cm^{-2}**	**Electrolyte**	**Reference**
**Ni/Fe-O(OH) on 3D nanostructures**	225	0.1 M KOH	This work
**Ni/Fe-O(OH) on Ni foam**	240	0.1 M KOH	(*24*)
**Ni/Fe-O(OH) on NiFe nanoparticles**	270	0.1 M KOH	(*28*)
**Fe-Doped β-Ni(OH)$_2$ on Ni foam**	219	1 M KOH	(*35*)

REFERENCES

1. Y. Wu, R. D. Tilley, J. J. Gooding, Challenges and solutions in developing ultrasensitive biosensors. *J. Am. Chem. Soc.* **141**, 1162–1170 (2019).

2. S. M. Moosavi, A. Nandy, K. M. Jablonka, D. Ongari, J. P. Janet, P. G. Boyd, Y. Lee, B. Smit, H. J. Kulik, Understanding the diversity of the metal-organic framework ecosystem. *Nat. Commun.* **11**, 4068 (2020).

3. P. W. K. Rothemund, Folding DNA to create nanoscale shapes and patterns. *Nature* **440**, 297–302 (2006).

4. L. Feng, K.-Y. Wang, G. S. Day, H.-C. Zhou, The chemistry of multi-component and hierarchical framework compounds. *Chem. Soc. Rev.* **48**, 4823–4853 (2019).

5. Q. Qian, Y. Li, Y. Liu, L. Yu, G. Zhang, Ambient fast synthesis and active sites deciphering of hierarchical foam-like trimetal–organic framework nanostructures as a platform for highly efficient oxygen evolution electrocatalysis. *Adv. Mater.* **31**, 1901139 (2019).

6. M. Grdeń, M. Alsabet, G. Jerkiewicz, Surface science and electrochemical analysis of nickel foams. *ACS Appl. Mater. Interfaces* **4**, 3012–3021 (2012).

7. A. Abelson, C. Qian, T. Salk, Z. Luan, K. Fu, J.-G. Zheng, J. L. Wardini, M. Law, Collective topo-epitaxy in the self-assembly of a 3D quantum dot superlattice. *Nat. Mater.* **19**, 49–55 (2020).

8. A. Dong, J. Chen, P. M. Vora, J. M. Kikkawa, C. B. Murray, Binary nanocrystal superlattice membranes self-assembled at the liquid–air interface. *Nature* **466**, 474–477 (2010).

9. H. Han, S. Kallakuri, Y. Yao, C. B. Williamson, D. R. Nevers, B. H. Savitzky, R. S. Skye, M. Xu, O. Voznyy, J. Dshemuchadse, L. F. Kourkoutis, S. J. Weinstein, T. Hanrath, R. D. Robinson, Multiscale hierarchical structures from a nanocluster mesophase. *Nat. Mater.* **21**, 518–525 (2022).

10. A. R. Poerwoprajitno, L. Gloag, J. Watt, S. Cheong, X. Tan, H. Lei, H. A. Tahini, A. Henson, B. Subhash, N. M. Bedford, B. K. Miller, P. B. O'Mara, T. M. Benedetti, D. L. Huber, W. Zhang, S. C. Smith, J. J. Gooding, W. Schuhmann, R. D. Tilley, A single-Pt-atom-on-Ru-nanoparticle electrocatalyst for CO-resilient methanol oxidation. *Nat. Catal.* **5**, 231–237 (2022).

11. B. Garlyyev, J. Fichtner, O. Piqué, O. Schneider, A. S. Bandarenka, F. Calle-Vallejo, Revealing the nature of active sites in electrocatalysis. *Chem. Sci.* **10**, 8060–8075 (2019).

12. J. Masa, C. Andronescu, W. Schuhmann, Electrocatalysis as the nexus for sustainable renewable energy: The Gordian knot of activity, stability, and selectivity. *Angew. Chem. Int. Ed.* **59**, 15298–15312 (2020).

13. A. R. Poerwoprajitno, S. Cheong, L. Gloag, J. J. Gooding, R. D. Tilley, Synthetic strategies to enhance the electrocatalytic properties of branched metal nanoparticles. *Acc. Chem. Res.* **55**, 1693–1702 (2022).

14. Y. Luo, Z. Zhang, M. Chhowalla, B. Liu, Recent advances in design of electrocatalysts for high-current-density water splitting. *Adv. Mater.* **34**, 2108133 (2022).

15. X. Lu, C. Zhao, Electrodeposition of hierarchically structured three-dimensional nickel–iron electrodes for efficient oxygen evolution at high current densities. *Nat. Commun.* **6**, 6616 (2015).

16. A. R. Poerwoprajitno, L. Gloag, J. Watt, S. Cychy, S. Cheong, P. V. Kumar, T. M. Benedetti, C. Deng, K. K.-H. Wu, C. E. Marjo, D. L. Huber, M. Muhler, J. J. Gooding, W. Schuhmann, D.-W. Wang, R. D. Tilley, Faceted branched nickel nanoparticles with tunable branch length for high-activity electrocatalytic oxidation of biomass. *Angew. Chem. Int. Ed.* **59**, 15487–15491 (2020).

17. L. Gloag, T. M. Benedetti, S. Cheong, Y. Li, X.-H. Chan, L.-M. Lacroix, S. L. Y. Chang, R. Arenal, I. Florea, H. Barron, A. S. Barnard, A. M. Henning, C. Zhao, W. Schuhmann, J. J. Gooding, R. D. Tilley, Three-dimensional branched and faceted gold-ruthenium

nanoparticles: Using nanostructure to improve stability in oxygen evolution electrocatalysis. *Angew. Chem. Int. Ed.* **57**, 10241–10245 (2018).

18. A. R. Poerwoprajitno, L. Gloag, T. M. Benedetti, S. Cheong, J. Watt, D. L. Huber, J. J. Gooding, R. D. Tilley, Formation of branched ruthenium nanoparticles for improved electrocatalysis of oxygen evolution reaction. *Small* **15**, 1804577 (2019).

19. M. Myekhlai, T. M. Benedetti, L. Gloag, V. R. Gonçales, S. Cheong, H. Chen, J. J. Gooding, R. D. Tilley, Increasing the formation of active sites on highly crystalline Co branched nanoparticles for improved oxygen evolution reaction electrocatalysis. *ChemCatChem* **12**, 3126–3131 (2020).

20. L. Gloag, T. M. Benedetti, S. Cheong, C. E. Marjo, J. J. Gooding, R. D. Tilley, Cubic-core hexagonal-branch mechanism to synthesize bimetallic branched and faceted Pd–Ru nanoparticles for oxygen evolution reaction electrocatalysis. *J. Am. Chem. Soc.* **140**, 12760–12764 (2018).

21. M. R. Buck, J. F. Bondi, R. E. Schaak, A total-synthesis framework for the construction of high-order colloidal hybrid nanoparticles. *Nat. Chem.* **4**, 37–44 (2011).

22. J. M. Hodges, A. J. Biacchi, R. E. Schaak, Ternary hybrid nanoparticle isomers: Directing the nucleation of Ag on Pt-Fe_3O_4 using a solid-state protecting group. *ACS Nano* **8**, 1047–1055 (2014).

23. J. Park, L. Zhang, S. Choi, L. T. Roling, N. Lu, J. A. Herron, S. Xie, J. Wang, M. J. Kim, M. Mavrikakis, Y. Xia, Atomic layer-by-layer deposition of platinum on palladium octahedra for enhanced catalysts toward the oxygen reduction reaction. *ACS Nano* **9**, 2635–2647 (2015).

24. H. Yang, L. Gong, H. Wang, C. Dong, J. Wang, K. Qi, H. Liu, X. Guo, B. Y. Xia, Preparation of nickel-iron hydroxides by microorganism corrosion for efficient oxygen evolution. *Nat. Commun.* **11**, 5075 (2020).

25. C. Zhou, S. Zhao, H. Meng, Y. Han, Q. Jiang, B. Wang, X. Shi, W. Zhang, L. Zhang, R. Zhang, $RuCoO_x$ nanofoam as a high-performance trifunctional electrocatalyst for rechargeable zinc–air batteries and water splitting. *Nano Lett.* **21**, 9633–9641 (2021).

26. S. Lentijo-Mozo, R. P. Tan, C. Garcia-Marcelot, T. Altantzis, P.-F. Fazzini, T. Hungria, B. Cormary, J. R. Gallagher, J. T. Miller, H. Martinez, S. Schrittwieser, J. Schotter, M. Respaud, S. Bals, G. van Tendeloo, C. Gatel, K. Soulantica, Air- and water-resistant noble metal coated ferromagnetic cobalt nanorods. *ACS Nano* **9**, 2792–2804 (2015).

27. L. Trotochaud, S. L. Young, J. K. Ranney, S. W. Boettcher, Nickel–iron oxyhydroxide oxygen-evolution electrocatalysts: The role of intentional and incidental iron incorporation. *J. Am. Chem. Soc.* **136**, 6744–6753 (2014).

28. C. Roy, B. Sebok, S. B. Scott, E. M. Fiordaliso, J. E. Sørensen, A. Bodin, D. B. Trimarco, C. D. Damsgaard, P. C. K. Vesborg, O. Hansen, I. E. L. Stephens, J. Kibsgaard, I. Chorkendorff, Impact of nanoparticle size and lattice oxygen on water oxidation on NiFeOxHy. *Nat. Catal.* **1**, 820–829 (2018).

29. F. Dionigi, P. Strasser, NiFe-based (oxy)hydroxide catalysts for oxygen evolution reaction in non-acidic electrolytes. *Adv. Energy Mater.* **6**, 1600621 (2016).

30. R. P. Forslund, W. G. Hardin, X. Rong, A. M. Abakumov, D. Filimonov, C. T. Alexander, J. T. Mefford, H. Iyer, A. M. Kolpak, K. P. Johnston, K. J. Stevenson, Exceptional electrocatalytic oxygen evolution via tunable charge transfer interactions in $La_{0.5}Sr_{1.5}Ni_{1-x}Fe_xO_{4\pm\delta}$ Ruddlesden-Popper oxides. *Nat. Commun.* **9**, 3150 (2018).

31. M. K. Bates, Q. Jia, H. Doan, W. Liang, S. Mukerjee, Charge-transfer effects in Ni-Fe and Ni-Fe-Co mixed-metal oxides for the alkaline oxygen evolution reaction. *ACS Catal.* **6**, 155–161 (2016).

32. S. Watzele, P. Hauenstein, Y. Liang, S. Xue, J. Fichtner, B. Garlyyev, D. Scieszka, F. Claudel, F. Maillard, A. S. Bandarenka, Determination of electroactive surface area of Ni-, Co-, Fe-, and Ir-based oxide electrocatalysts. *ACS Catal.* **9**, 9222–9230 (2019).

33. K. Fominykh, J. M. Feckl, J. Sicklinger, M. Döblinger, S. Böcklein, J. Ziegler, L. Peter, J. Rathousky, E.-W. Scheidt, T. Bein, D. Fattakhova-Rohlfing, Ultrasmall dispersible crystalline nickel oxide nanoparticles as high-performance catalysts for electrochemical water splitting. *Adv. Funct. Mater.* **24**, 3123–3129 (2014).

34. E. Cossar, A. Oyarce Barnett, F. Seland, E. A. Baranova, The performance of nickel and nickel-iron catalysts evaluated as anodes in anion exchange membrane water electrolysis. *Catalysts.* **9**, 814 (2019).

35. T. Kou, S. Wang, J. L. Hauser, M. Chen, S. R. J. Oliver, Y. Ye, J. Guo, Y. Li, Ni foam-supported Fe-doped β-$Ni(OH)_2$ nanosheets show ultralow overpotential for oxygen evolution reaction. *ACS Energy Lett.* **4**, 622–628 (2019).
